# An across-breed validation study of 46 genetic markers in canine hip dysplasia

**DOI:** 10.1186/s12864-021-07375-x

**Published:** 2021-01-21

**Authors:** Lea Mikkola, Kaisa Kyöstilä, Jonas Donner, Anu K. Lappalainen, Marjo K. Hytönen, Hannes Lohi, Antti Iivanainen

**Affiliations:** 1 Department of Veterinary Biosciences, University of Helsinki, P.O. BOX 66 (Agnes Sjöbergin katu 2), 00014 Helsinki, Finland; 2 Department of Medical and Clinical Genetics, University of Helsinki, Helsinki, Finland; 3 Folkhälsan Research Centre, Helsinki, Finland; 4 Wisdom Health, Helsinki, Finland; 5 Department of Equine and Small Animal Medicine, University of Helsinki, Helsinki, Finland

**Keywords:** Hip dysplasia, Dog, Canine, GWAS, Validations study, Neddylation

## Abstract

**Background:**

Canine hip dysplasia (CHD) is a common disease, with a complex genetic background. Dogs with severe CHD sometimes also suffer from osteoarthritis (OA), an inflammatory, often painful and incurable condition. Previous studies have reported breed-specific genetic loci associated with different hip dysplasia and OA phenotypes. However, the independent replication of the known associations within or across breeds has been difficult due to variable phenotype measures, inadequate sample sizes and the existence of population specific variants.

**Results:**

We execute a validation study of 46 genetic markers in a cohort of nearly 1600 dogs from ten different breeds. We categorize the dogs into cases and controls according to the hip scoring system defined by the Fédération Cynologique Internationale (FCI). We validate 21 different loci associated on fourteen chromosomes. Twenty of these associated with CHD in specific breeds, whereas one locus is unique to the across-breed study. We show that genes involved in the neddylation pathway are enriched among the genes in the validated loci. Neddylation contributes to many cellular functions including inflammation.

**Conclusions:**

Our study successfully replicates many loci and highlights the complex genetic architecture of CHD. Further characterisation of the associated loci could reveal CHD-relevant genes and pathways for improved understanding of the disease pathogenesis.

**Supplementary Information:**

The online version contains supplementary material available at 10.1186/s12864-021-07375-x.

## Background

Revealing the genetic background of canine hip dysplasia (CHD) has remained one of the biggest veterinary conundrums in the past few decades. Although there has been a lot of effort to uncover risk loci and causal variants, their validation and replication has proven difficult. An inadequate sample size has been an issue in many studies but studies have also been hampered by the complexity and inaccuracy of the phenotypes, and an apparent genetic heterogeneity across breeds.

The prevalence of CHD is highly variable between breed groups and individual breeds. For example, in USA and Canada, where dogs scored within the American Veterinary Medical Association system, the extreme values vary from a zero disease prevalence in Italian Greyhounds to a high prevalence of 77.7% in the Bulldogs [1]. Consequently, it is important to find both the genetic factors that are breed-specific and those that are shared between breeds. Validation of breed-specific loci is a tedious effort, since one needs to collect a robustly phenotyped independent cohort of each breed. It is usually easier to collect a large multi-breed cohort that can be used to define which CHD associated loci are shared between breeds. The accuracy of the studied phenotypes is imperative. The Finnish Kennel Club (FKC) implements the hip scoring system defined by the Fédération Cynologique Internationale (FCI) [2], and uses only few specialised veterinarians to evaluate the hip scores, therefore reducing the inter-observer bias [3].

The single nucleotide polymorphic genetic markers (SNPs) evaluated in this study have been associated with CHD by us and others over the past ten years. Zhou et al. (2010) identified a total of six SNPs in their association study of Norberg angle (a measure of hip joint laxity) and OA in several breeds [4]. Friedenberg et al. (2011) found a homozygous deletion haplotype (intronic to Fibrillin 2; *FBN2*) associated with a severe form of CHD in Labrador Retrievers, as well as in 14 other breeds and in a cross-bred (Labrador Retriever - Greyhound) dog cohort [5]. Pfahler and Distl (2012) conducted a genome-wide association study (GWAS) of the FCI hip score in Bernese Mountain dogs and found three significantly associated SNPs representing two different loci [6]. Fels and Distl (2014) carried out a validation study with the FCI hip score in the German Shepherd [7]. They reported three significantly associated SNPs from canine chromosomes CFA24, CFA26, and CFA34 [7]. Fels et al. (2014) uncovered novel SNPs in previously identified quantitative trait loci (QTL) and found that nine SNPs in five loci associated significantly with the FCI hip score in their German Shepherd cohort [8]. Lavrijsen et al. (2014) used GWAS and exon sequencing to identify multiple alleles associated with the FCI hip score in Dutch Labrador Retrievers [9]. Sanchez-Molano et al. (2014) also studied Labrador Retrievers using a hip score defined by the British Veterinary Association and the Kennel Club; they revealed two significantly associated QTL including several SNPs [10]. Bartolome et al. (2015) reported a genetic predictive model for CHD based on seven SNPs they found using GWAS and candidate gene approaches in Labrador Retrievers [11]. Additional markers were provided by our collaborator at Genoscoper Laboratories [12]. Our own previous case-control GWAS of the FCI hip score in German Shepherds [13] revealed loci on CFA1 and CFA9 that harboured markers with either risk or protective alleles.

This study aimed to validate the previously reported associations to better understand their significance and overall genetic heterogeneity of CHD. We selected 52 SNPs based on prior research, and carried out across- and within breeds replication studies in nearly 1600 dogs from 10 breeds. We successfully replicated five markers across breeds and identified 20 markers in different breeds, which highlights the heterogeneous genetic architecture of CHD.

## Results

We genotyped 52 SNPs that have previously been associated with CHD (Additional files 1 and 2), and carried out various case-control association analyses of CHD using an independent cohort of 1607 dogs consisting of 10 breeds. After quality control 46 SNPs and 1570 dogs (751 cases and 819 controls, 666 males and 904 females) remained for our analyses. The variant data of the 46 SNPs in our cohort are available through the European Variation Archive (https://www.ebi.ac.uk/eva). These SNPs are referred to by their ssIDs in this paper (see Additional file 1). We used the FCI hip score to categorize dogs into cases (hip score C or worse on both joints) and controls (hip score A/A). Dogs with hip score B were excluded to minimize phenotype ambiguity because although considered normal the FCI hip score B represents a borderline normal phenotype. We used the Cochran-Mantel-Haenszel 2x2xK (CMH) test for the breed-stratified data in our analyses.

### Four SNPs associated with CHD across breeds

The raw *P*-values, the empirical P-values from the permutation procedure, and odds ratios (OR) from the across-breed CMH test as implemented in PLINK [14] are shown in Table 1. Only the markers with a significant association to CHD, and which also passed a test of homogeneity of OR across the breeds (see Methods), are reported. A total of four SNPs associated significantly with CHD in the across-breed analysis (Table 1). The markers are located on CFA1, CFA14, CFA26, and CFA37. Of these, the SNP on CFA1 is from our previous study of the FCI hip score on German Shepherds [13]. The variant ss7212922135 on CFA14 was originally associated with the FCI hip score in Bernese Mountain dogs [6]. On CFA26, ss7212922151 originally associated with the FCI hip score in German Shepherds [7], as did ss7212922122 on CFA33 [8]. The variant ss7212922139 on CFA37 originally associated with OA in a multi-breed analysis [4]. In contrast to the other four markers, ss7212922139 in CFA37 was not significantly associated with CHD in any of the breed-specific analyses.

**Table 1:** SNPs demonstrating significant association to CHD in the across-breed CMH test for stratified data

SNP	Location in CanFam3.1	Allele (minor / major)	Raw ***P***-value (P_**RAW**_)	Permuted P-value (P_**GEN**_)	OR (95% CI)	Origins of the SNP (study, breeds, and phenotypes)
ss7212922120	CFA1: 45382633	A/C	7.4 × 10–03	8.4 × 10–03	1.30 [1.07–1.57]	Mikkola et al. (2019) [13]/ German Shepherd / FCI hip score
ss7212922135	CFA14: 56572744	T/C	0.027	0.028	1.19 [1.02–1.38]	Pfahler & Distl (2012) [6]/ Bernese Mountain dog / FCI hip score
ss7212922151	CFA26: 14157064	T/C	8.8 × 10–03	0.011	1.26 [1.06–1.49]	Fels & Distl (2014) [7]/ German Shepherd/ FCI hip score
ss7212922139	CFA37: 14299531	C/A	0.030	0.032	0.84 [0.72–0.98]	Zhou et al. (2010) [4]/ Multiple breeds and breed crosses/ OA

*CI* confidence interval; *OR* odds ratio. The odds ratios and their 95% confidence intervals were calculated by PLINK using default settings in which the minor allele increases the risk when OR > 1. The reference genome used in this study is CanFam3.1

### Within-breed analyses reveal additional markers

The definition of cases and controls was the same as it was in the across-breed analysis. We used two methods, basic association analysis by X^2^-test of allele frequencies and logistic regression, to assess breed-specific association of the SNPs to CHD. The analysis method that demonstrated a better model fit, as evaluated by visual examination of quantile-quantile (Q-Q) plots (see Additional file 3), was chosen for each breed. Logistic regression showed better model fit for five breeds and the basic association test for the other five (see Additional file 3). Some inflation of the expected versus observed *P*-values was observed in three breeds: Finnish Lapphund, Golden Retriever, and Labrador Retriever (see Additional file 3). All these breeds have at least partially separated breeding lines of herding/working dogs (meaning restricted mixing of the breeding dogs between lines), which may be the source for the inflation.

The within-breed analyses showed multiple significant associations for different SNPs per breed (Table 2). The number of significantly associated SNPs varied between breeds, ranging from none in Bernese Mountain dogs to six significant associations in the Labrador Retrievers (Table 2). In total, 22 SNPs on CFA1, CFA3, CFA8, CFA11, CFA12, CFA14, CFA17, CFA21, CFA24, CFA25, CFA26, CFA33, and CFA34 demonstrated significant associations to CHD (Table 2). Six SNPs were significant in more than one breed (ss7212922122, ss7212922151, ss7212922154, ss7212922155, ss7212922161, ss7212922163 Table 2). These six SNPs are located on CFA33, CFA26, CFA34, CFA11, CFA1, and CFA24. The marker ss7212922155 on CFA11 was significant on three breeds (Table 2).

**Table 2:** SNPs demonstrating significant association with CHD in nine^d^ different breeds

Breed (N in analysis)	SNPs with significant associations with CHD	Location in CanFam3.1	Reference	Allele (minor / major)	OR (95% CI)	P-values: raw / permuted
Finnish Lapphund(303)	HD_25_51006290 ss7212922151 ss7212922146 ss7212922122 ss7212922154	CFA25: 47983231 CFA26: 14157064 CFA33: 4052295 CFA33: 7967486 CFA34: 11240092	[12] [7] [8] [8] [8]	G/A T/C C/T C/T A/G	5.56 [1.22–25.28] 1.63 [1.14–2.33] 1.50 [1.09–2.07] 0.68 [0.49–0.94] 0.67 [0.48–0.92]	0.013 / 0.021 ^a^ 6.8 × 10–03 / 9.7 × 10–03 ^a^ 0.013 / 0.012 ^a^ 0.019 / 0.021 ^a^ 0.015 / 0.019 ^a^
Golden Retriever(235)	ss7212922155 ss7212922147 ss7212922135	CFA11: 54489194 CFA12: 16410934 CFA14: 56572744	[4] [11] [6]	G/A A/G T/C	0.57 [0.37–0.89] 0.56 [0.33–0.97] 1.55 [1.02–2.35]	0.014 / 0.013 ^b^ 0.037 / 0.033 ^b^ 0.040 / 0.034 ^b^
Lagotto Romagnolo(194)	ss7212922120 ss7212922155 ss7212922151	CFA1: 45382633 CFA11: 54489194 CFA26: 14157064	[13] [4] [7]	C/A^c^ G/A T/C	0.59 [0.36–0.95] 0.60 [0.38–0.94] 2.00 [1.08–3.71]	0.029 / 0.028 ^a^ 0.025 / 0.040 ^a^ 0.025 / 0.028 ^a^
Samoyed(149)	ss7212922144 ss7212922163	CFA3: 40302288 CFA24: 25973438	[11] [7]	A/G C/G	0.47 [0.28–0.78] 1.75 [1.06–2.90]	3.2 × 10–03 / 7.3 × 10–03 ^a^ 0.029 / 0.033 ^a^
Spanish Water dog(114)	ss7212922131 ss7212922122	CFA21: 40146141 CFA33: 7967486	[10] [8]	C/T C/T	0.51 [0.29–0.89] 0.48 [0.25–0.90]	0.017 / 0.014 ^b^ 0.023 / 0.022 ^b^
Great Dane(113)	ss7212922137 ss7212922133	CFA1: 46279297 CFA14: 20864104	[13] [6]	G/A C/G	2.14 [1.15–3.97] 5.07 [1.04–24.73]	0.016 / 0.017 ^b^ 0.045 / 0.036 ^b^
Labrador Retriever(100)	ss7212922118 ss7212922126 ss7212922153 ss7212922141 ss7212922163 ss7212922154	CFA1: 45161186 CFA1: 67883139 CFA1: 67942900 CFA11: 29908777 CFA24: 25973438 CFA34: 11240092	[13] [9] [9] [4] [7] [8]	C/G A/T T/A A/T G/C G/A	1.83 [1.01–3.29] 2.51 [1.09–5.74] 2.55 [1.11–5.86] 0.47 [0.25–0.87] 1.87 [1.07–3.28] 0.52 [0.27–0.97]	0.046 / 0.037 ^b^ 0.030 / 0.023 ^b^ 0.027 / 0.020 ^b^ 0.016 / 0.017 ^b^ 0.029 / 0.023 ^b^ 0.040 / 0.027 ^b^
Karelian Bear dog(97)	ss7212922161 ss7212922156 ss7212922152 ss7212922155	CFA1: 67876393 CFA8: 28489063 CFA8: 28489546 CFA11: 54489194	[12] [12] [12] [4]	A/C T/C A/C A/G	0.46 [0.23–0.91] 0.41 [0.17–0.96] 0.41 [0.17–0.96] 2.94 [1.41–6.10]	0.025 / 0.019 ^b^ 0.041 / 0.035 ^b^ 0.041 / 0.035 ^b^ 3.9 × 10–03 / 4.3 × 10–03 ^b^
Finnish Hound (102)	ss7212922123	CFA17: 45022358	[4]	T/G	2.38 [1.31–4.34]	4.1 × 10–03 / 8.7 × 10–03 ^a^

^a^X^2^-test. ^b^Logistic regression. *CI* confidence interval; *OR* odds ratio. The odds ratios and their confidence intervals were calculated by PLINK using default settings in which the minor allele increases the risk when OR > 1. The reference genome used in this study is CanFam3.1. ^c^No significant associations were observed in Bernese Mountain dogs. ^d^Major/minor allele designation of ss7212922120 is changed in Lagotto Romagnolo cohort

ss7212922126 and ss7212922153 on CFA1, as well as ss7212922156 and ss7212922152 on CFA8 were in high linkage disequilibrium (r^2^ > 0.80) and therefore these SNP pairs were interpreted to represent one locus each. Thus, the 22 markers associating with CHD and with OR’s deviating from 1 represent 20 different loci on 13 chromosomes (Table 2).

### Enrichment of the neddylation-pathway

**Fig. 1:**
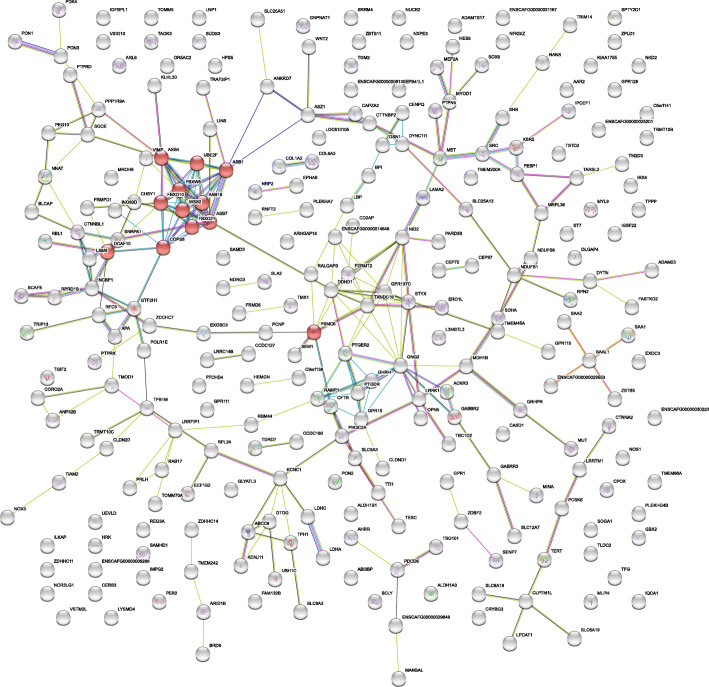
The protein network of the positional candidate genes from all of the 21 loci. Each circle represents one protein. Red circles indicate proteins that belong to the neddylation pathway. The lines between circles indicate the evidence for the association between proteins. The thicker the line, the stronger the evidence. The total number of nodes is 263. For the complete STRING analysis, see https://version-11-0.string-db.org/cgi/network.pl?networkId=5Sqk4IV9gi5b

Our across and within breed analyses highlighted altogether 21 loci on fourteen chromosomes. These loci contain hundreds of candidate genes and we wanted to understand whether they are enriched in any cellular pathways. We performed two analyses using the Search Tool for the Retrieval of Interacting Genes/Proteins (STRING) searching for the dog genes/proteins [15], including all the positional candidate genes within 1 Mb of the associated SNPs. In the first analysis, no enriched pathways were detected among the 58 positional candidate genes from the across-breed study (see Additional File 4). For the second analysis, we pooled all the 272 positional candidate genes from the across and within breed studies (Additional File 4). An enrichment in the neddylation pathway (Reactome ID: CFA-8951664) was spotted by STRING with a false discovery rate of 0.0314 (12 observed genes / 220 genes in the neddylation-pathway gene set, see Fig. 1 and Table 3).

**Table 3:** Enriched candidate genes in the neddylation-pathway

Gene	Location in CanFam3.1	Position of the associated marker(s) within the chromosome (locus; distance from the gene)
*ASB7*	CFA3: 40365515–40415345	CFA3: 40302288; 63.227 kb
*PSMC6*	CFA8: 28914302–28947854	CFA8: 28489064; 425.238 kb CFA8: 28489547; 424.755 kb
*FBXO10*	CFA11: 53833335–53885416	CFA11: 29908777; 23,924.558 kb CFA11: 54489194; 603.778 kb
*DCAF10*	CFA11: 54107165–54150058	CFA11: 29908777; 24,198.388. kb CFA11: 54489194; 339.136 kb
*ASB4*	CFA14: 20733592–20794394	CFA14: 20864104; 69.710 kb CFA14: 56572744; 35,778.350 kb
*ASB18*	CFA25: 47026112–47087245	CFA25: 47983231; 895.986 kb
*COPS8*	CFA25: 47783241–47796806	CFA25: 47983231; 186.425 kb
*UBE2F*	CFA25: 48503866–48558950	CFA25: 47983231; 520.635 kb
*ASB1*	CFA25: 48834475–48870290^a^	CFA25: 47983231; 851.244 kb
*FBXW8*	CFA26: 13520605–13640975	CFA26: 14157064; 516.089 kb
*FBXO21*	CFA26: 13722301–13769273	CFA26: 14157064; 387.791 kb
*WSB2*	CFA26: 14440035–14456158	CFA26: 14157064; 282.971 kb

^a^ASB1 coordinates were derived from the Ensembl database (ENSCAFG00000012461, https://www.ensembl.org). Other coordinates were retrieved from the National Center for Biotechnology Information database (NCBI)

## Discussion

Previous efforts by us and others have discovered tens of loci associating with CHD across breeds. However, the significance of these findings has remained vague due to the lack of proper replication because of the variability of the phenotypes, varied study approaches and inadequate sample sizes.

Our replication study in over 1600 dogs across 10 breeds with 52 reported CHD markers validates 21 loci on fourteen chromosomes. Five loci were associated across breeds. Two loci included more than one validated marker. Inclusion of more markers in the study would have made the validation of each locus more robust and possibly allowed further selection of associated loci by the presence of clusters of validated SNPs. Also, after our analyses several interesting markers were published [16].

In total, the replicated loci include over 250 genes with an enrichment of candidate genes in a neddylation pathway. Neddylation contributes to various cellular functions, including inflammation, commonly found in CHD and OA. Collectively, these results highlight the complex genetic background of CHD and provide important insights to the significance of the common and breed-specific loci in CHD. This helps to prioritize loci for further studies to identify causal variants and suggest a novel hypothesis for the possible contribution of the affected neddylation pathway to CHD and OA.

Four loci on CFA1, CFA14, CFA26, and CFA37 associated with CHD across breeds. The variants ss7212922118 and ss7212922120 on CFA1 were reported by us to associate with CHD in German Shepherds [13]. These SNPs locate within and upstream of *NADPH Oxidase 3* (*NOX3*), which is a catalyst for the formation of superoxides and other reactive oxygen species. NADPH oxidases are an essential part of a reaction chain that has been suggested to contribute to the initiation of articular cartilage degradation [17]. However, *NOX3* is mainly expressed in the inner ear and foetal tissues, which leaves its role in CHD equivocal.

The SNP on CFA14 (ss7212922135) was originally reported to associate with CHD in Bernese Mountain dogs [6]. This SNP did not associate with CHD in our Bernese Mountain dog cohort, which may result from different case definition between the current and the earlier study. The variant ss7212922135 originated from a study cohort where all cases (*N* = 33) had mild CHD (FCI score C) [6], while our Bernese Mountain dog cases represented mild-to-severe CHD based on FCI hip scoring (N_cases_ = 88; 42 with mild (FCI score C), 40 with moderate (FCI score D), and six with severe (FCI score E) CHD). However, ss7212922135 was significant in the across-breed analysis. This SNP lies within the ninth intron of Cortactin Binding Protein 2 encoding gene (*CTTNBP2*) [6]. CTTNBP2 participates in brain development and the regulation of synapse organisation. Although no obvious connection was found between this gene and CHD in the original study [6], some more recent phenotype associations have been listed in the GWAS catalogue (https://www.ebi.ac.uk/gwas/home) for *CTTNBP2* that are worth noting: juvenile idiopathic arthritis and idiopathic osteonecrosis of the femoral head. Furthermore, body mass index adjusted waist-hip ratio, a measure for storage fat in humans, is listed in the GWAS catalogue [18] for *ST7*, *WNT2*, *ASZ1*, and *CFTR*, all within ±1 Mb of ss7212922135. Obesity is a known environmental risk factor for hip dysplasia and OA in both dogs and humans [19], although the mechanism is still poorly understood. Finally, we want to highlight the gene encoding Wnt Family Member 2 (*WNT2*) ~ 401 kb downstream ss7212922135. Wnt signalling pathways have been shown to participate in joint development, cartilage maintenance homeostasis, and the development and progression of OA [20].

Variant ss7212922151 on CFA26 was the third SNP, which demonstrated significant association to CHD in the across-breed analysis. This SNP originates from an association study of CHD in German Shepherds and locates within the fifth intron of Kinase Suppressor Of Ras 2 encoding gene (*KSR2*) [7]. KSR2, a scaffolding protein in the Ras-Raf-MEK-ERK pathway, has been associated with obesity in mice and humans [21, 22]. Although the non-SMAD-dependent TGF-β/BMP signalling in the osteoblastic lineage involves MKK3/6 and p38 [23], we are not aware of any studies reporting on their interaction with KSRs. Another gene of interest in the same locus is *Nitric Oxide Synthase 1* (*NOS1*; ~ 262 kb away from ss7212922151). A large variety of different phenotypes have been reported in *Nos1* murine models, including abnormal skeletal and skeletal muscle phenotypes for *Nos1*^*tm1Plh*^-mutants [24].

ss7212922122 on CFA33 locates within the gene encoding PEST Proteolytic Signal Containing Nuclear Protein (*PCNP*), and was originally found to associate with CHD in German Shepherds [8]. PCNP expression is omnipresent in different tissues, and with its ubiquitination partner Np95/ICBP90-like RING finger protein (NIRF) it may be involved in a signalling pathway of cell cycle regulation and/or genome stability [25, 26]. ss7212922122 is also about 469 kb upstream from *ABI Family Member 3 Binding Protein* (*ABI3BP*), which is a collagen and glycosaminoglycan binding molecule and an extracellular matrix structural component. Moreover, an intron variant of *ABI3BP* (rs9828061) has been associated with joint hypermobility measurement in humans [27].

Lastly, ss7212922139 on CFA37 locates within the first intron of *Neuropilin 2* (*NRP2*). It was the only marker that despite not being significantly associated with CHD in any particular breed was still significantly associated with CHD in the across-breed analysis. Indeed, this SNP originated from a study, which utilised association and linkage populations of multiple breeds and their crosses [4]. In our study the marker had OR < 1, indicating protective effect. The original study did not report ORs, and the SNP associated with OA [4]. Although the current study data did not contain the direct OA phenotypes, OA is nevertheless assessed and considered when determining the FCI score. Considering the original and current study, ss7212922139 could represent a genuine “across-breed locus” for CHD and OA. Zhou et al. (2010) suggested *Par-3 Family Cell Polarity Regulator Beta* (*PARD3B*) as a candidate gene due to its association with OA of the knee in humans [4, 28]. In addition to *PARD3B*, *NRP2* could also be a plausible candidate for OA. Neuropilin 2 is as a co-receptor for vascular endothelial growth factors (VEGFs) [29]. Increased VEGF expression has been indicated to associate with increased severity of OA [30], and a functional study demonstrated that VEGF injections into knee joints of mice induced OA [31]. Moreover, VEGF and its receptors have been studied as targets for treatment of OA [32], and an experimental study of a VEGF antibody (rhu-Mab-VEGF; Bevacizumab or Avastin®) has shown that Bevacizumab could offer a potential therapy for OA [33]. However, it remains unknown how NRP2-VEGF interaction could affect the development and progression of OA in dogs.

The current within-breed analyses revealed a multitude of loci that associated with CHD, strengthening the hypothesis that CHD has a complex genetic architecture and distinct genetic backgrounds in different breeds. The highest number of associated SNPs was found with Labrador Retriever (6), while none of the tested markers associated with CHD in our Bernese Mountain dog cohort. The associated markers and loci varied between breeds. Some SNPs demonstrated association to CHD in more than one breed and ss7212922151 on CFA26 was also significant in the across-breed analysis. We want to acknowledge here that the results in the Finnish Lapphund, Golden Retriever and Labrador Retriever breeds are inflated due to possible population stratification (as evidenced by the Q-Q plots, see Additional file 3). These breeds have both show and working/herding lines, which we could not account for in this study due to the missing line information. Thus, the breed-specific results for these three breeds should be regarded with some caution.

Some of the significant markers from the within-breed analyses had notably higher or lower ORs (Tables 1 and 2), which is probably due to higher across-breed variation for these markers. Also, worth noting was that some breeds had ORs of over 5 for certain markers (Table 2), which indicates relatively strong association to the disease outcome for such a complex disorder. Future studies should concentrate on these loci for breeds such as the Great Dane that had OR of 5.07 for ss7212922133 on CFA14, and had no marked inflation observed (see Additional file 3, plots M and N).

This study replicated altogether 21 loci with over 250 potential candidate genes raising an interesting question of the possible relationship and enrichment of the candidate genes in the associated loci predisposing to CHD. The STRING analysis revealed that the candidate genes were enriched in a single pathway, neddylation, which is a ubiquitination-like conserved post-translational protein modification process [34]. The key actor in neddylation is NEDD8, which is conjugated to its substrates with the help of enzymes E1 (activation of NEDD8; NAE1-UBA3 heterodimer), E2 (conjugation; UBE2M or UBE2F), and E3 (ligase; RBX1 or RBX2) [35]. Neddylation modifies the biochemical properties of the target substrates [34], such as many members of the cullin-family, p53, or EGFR [35, 36]. Neddylation is essential for cell cycle progression [37, 38], and it has been linked to many pathologies, especially to human cancers [36, 37]. Neddylation was recently linked to inflammatory arthritis via increased NF-κB activation, although increased expression of neddylation-related genes (NEDD8 and CUL1) were observed only in the synovium of the rheumatic arthritis and not in the controls with non-inflammatory OA [39].

Interestingly, searching the STRING database with the 12 neddylation pathway associated candidate genes from the current study (Table 3) and with 14 genes (*CYBA, MAPK14, MMP2, MMP9, NCF1, NCF2, NCF4, NOX3, NOXA1, NTN1, RAC1, RAC1, TRIO, VCAM1*) highlighted in our previous studies on CHD on German Shepherds [13, 40] produced two gene clusters that shared 12 genes associated with Class I MHC mediated antigen processing & presentation (R-CFA-983169, https://version-11-0b.string-db.org/cgi/network?networkId=bPwtieGk6WuV, and Additional file 5).

There is cumulating evidence that inflammatory mechanisms are active in OA [41, 42]. Neddylation is demonstrated to participate in regulation of i.a. T-cell and macrophage functions during inflammation [39, 43, 44]. Both T-cell and macrophage mediated inflammatory responses have been observed in OA [42, 45], and therefore, the possible role of neddylation-pathway in the immune base of OA should be explored further.

## Conclusions

We replicate 21 previously reported CHD-associated loci on fourteen chromosomes and identify neddylation as a novel candidate pathway for CHD and OA. We identify common and breed-specific loci and highlight the complex genetic architecture of CHD. Identification of the causal genes and variants in the associated loci remains as an important future task to better understand the molecular pathogenesis of CHD and its subtraits towards improved treatment and diagnostic options.

## Methods

### Study cohorts and phenotype

We used SNP genotyping to validate 52 SNPs on several chromosomes in a large cohort comprising of ten breeds. Our cohort consisted of 1607 dogs with FCI hip scores (Table 4), which were used to categorize dogs into cases (hip score C or worse on both joints, *N* = 772) and controls (hip score A/A, *N* = 835). It is worth noting that the FCI scoring does not represent a quantitative phenotype. Furthermore, the distribution of phenotype categories of the cohort is very skewed and does not meet the assumptions required for the analysis of datasets in the ordinary scale. Because of these reasons, only case-control analysis was possible. To minimize phenotype ambiguity, dogs with hips scored B were excluded. Although score B is considered normal, it nevertheless represents a borderline normal phenotype.

**Table 4:** Number of dogs and SNPs per breed before and after quality control

Breed	Number of dogs before breed-specific QC (N cases/N controls)	Number of dogs after breed-specific QC (N cases/N controls)	SNPs after breed-specific QC
Finnish Lapphund	307 (155/152)	303 (153/150)	48
Golden Retriever	244 (84/160)	236 (79/156)	47
Lagotto Romagnolo	200 (100/100)	194 (96/98)	48
Bernese Mountain dog	172 (94/78)	164 (89/75)	48
Samoyed	150 (72/78)	149 (71/78)	48
Spanish Water dog	114 (56/58)	114 (56/58)	49
Great Dane	114 (57/57)	113 (57/56)	50
Labrador Retriever	102 (55/47)	100 (54/46)	48
Karelian Bear dog	102 (49/53)	97 (47/50)	49
Finnish Hound	102 (50/52)	102 (50/52)	49
**Total quantity**	1607 (772/835)	1572 (753/819)	–

The participating breeds were (in order of number of samples per breed): Finnish Lapphund, Golden Retriever, Lagotto Romagnolo, Bernese Mountain dog, Samoyed, Spanish Water dog, Great Dane, Labrador Retriever, Karelian Bear dog, and Finnish Hound. These breeds were chosen for the project because the prevalence of CHD in them is at least moderate. FKC collects FCI hip scoring data into an open-access breeding database from which the phenotypes were gathered for this study [2, 46]. We investigated the prevalence of CHD in the above-mentioned breeds as the mean of observed yearly prevalences including all cases from FCI hip score C to E, measured in an eleven-year period (2006–2016, year of birth) [2, 46]. The lowest mean prevalence was observed for Labrador Retriever (19%) and the Finnish Hound (28%) [46]. Finnish Lapphund, Golden Retriever, Samoyed, Spanish Water dog, and Great Dane all had a mean prevalence of CHD between 30 and 40% during this time period [46]. The highest mean prevalence was observed for the Karelian Bear dog (41%), Bernese Mountain dog (43%), and Lagotto Romagnolo (45%) [46].

To minimise possible genomic stratification and subsequent inflation of the test statistics we were careful not to incorporate close relatives and included only one individual from all core-families during the initial data collection. Nevertheless, more distant relatedness may exist within the breeds. Also, Finnish Lapphund, Golden Retriever and Labrador Retriever are breeds that have working/herding breeding lines, which are at least partially separate from the rest of the breeding dogs. In the current study we did not have the breeding line information for these dogs and could not account for it in the analyses. This may cause additional inflation of the test statistics due to stratification within the breed. The breed-specific results for these three breeds must therefore be interpreted with some caution.

### SNP genotyping

Agena MassARRAY® iPLEX was used to genotype 52 SNPs (Additional files 1 and 2) in 1607 dogs from ten breeds. Six of the SNPs were chosen for the project from our own study in German Shepherds [13]. Ten markers from CFA1, CFA5, CFA8, CFA20, and CFA25 were selected based on investigations by Wisdom Health. These markers are included in a patent (Patent no.: US10150998B2 [12]) and five of them were also in Lavrijsen et al. (2014) [9]. The rest of the markers were chosen from earlier studies of Zhou et al. (2010) [4], Friedenberg et al. (2011) [5], Pfahler and Distl (2012) [6], Bartolome et al. (2015) [11], Fels and Distl (2014) [7], Fels et al. (2014) [8], and Sanchez-Molano et al. (2014) [10] to evaluate whether results were replicable in our data set. Data from the previous studies (breed, size of cohort and reported raw and corrected *P*-values) are summarized for each marker in Additional file 1.

The genotyping was performed by the Institute of Molecular Medicine Finland (FIMM) Technology Centre, University of Helsinki. The genotyping was done in two separate batches; the first batch included samples from breeds Labrador Retriever, Golden Retriever, and Bernese Mountain Dog; the second batch included samples from breeds Spanish Water Dog, Karelian Bear Dog, Lagotto Romagnolo, Finnish Hound, Samoyed, Finnish Lapphund, and Great Dane. Initial quality control of data was carried out in FIMM. In the first batch one sample was discarded due to success rates lower than 70%, and four SNP assays were discarded due to unreliable or no results. In the second batch, five samples were discarded due to success rates lower than 70%, and two SNP assays were discarded due to unreliable or no results. The resulting data were delivered to us as map and ped files. The map file was based on the CanFam3.1. reference, Annotation Release 104.

### Quality control

We carried out the quality control in stages for within-breed and across-breed association analyses. Initially there were 1607 samples and 52 SNPs before the QC steps. The QC thresholds for the breed-specific data were: 0.90 for per ID and per SNP call rates, 0.05 for the minor allele frequency (MAF), and 0.0001 for the cut-off *p*-value in check for Hardy-Weinberg Equilibrium (HWE) (done in controls only). The resulting breed-specific data is described in Table 4. SNP-specific failures to meet the QC criteria are presented in Additional file 1.

The QC for the across-breed data was done in two steps in PLINK. The first QC step was done at the breed-level before merging, with the following thresholds: 0.90 for per ID and per SNP call rates, 0.0001 for the cut-off p-value in check for HWE check in controls. MAF cut-off level was set to zero in this initial step, because some SNPs that might not pass the MAF threshold within a breed, might however pass it in the across-breed cohort. Subsequently, the SNPs and individuals that passed this initial QC step were merged into the across-breed data set. The final QC with the MAF cut-off threshold at 0.05 was then executed over the whole across-breed data, which left us 46 SNPs and 1572 samples. However, one Bernese Mountain dog and one Golden Retriever had hip scores of B/D (left hip/right hip) and they were excluded from the analysis, because we could not rule out the possibility of unilateral CHD induced by an injury or other environmental factor. Therefore, we finally had 1570 dogs in our analyses, of which 751 were cases, 819 were controls, and 666 were males and 904 were females.

### Association analysis

Before the analyses we tested the quality-controlled SNP data for possible batch effects in R with the glm-function. This was done because the genotyping was executed in two batches. We did not observe any significant batch effects. The within-breed analyses were carried out with the --assoc (X^2^-test of allele frequencies) or with --logistic (logistic regression) functions in PLINK, with age at radiographing as covariate in the logistic model (--assoc cannot use covariates).

As the SNPs in the study are a strongly selected and small subset of all the SNPs in the genome, the quantification of inflation is a challenge. QQ-plots were used to assess the overall fit of the alternative models and to select between them (Additional file 3). The QQ-plot is a more representative proxy for the fit of the model than the lambda value by PLINK which is based on the ratio of single (median) value of the test variable.

Some inflation of the test statistics in both analysis methods was observed in three breeds (see Additional file 3, plots A–D and O–P): Finnish Lapphund, Golden Retriever, and Labrador Retriever. We did not attempt to quantify or control the stratification in these breeds by PCA as it is not expected to work when the number of markers is small and the effect of any single marker is expected to be low [47] as is the case in our study.

Odds ratios and their 95% confidence intervals were calculated in PLINK using the default settings and the function --ci. PLINK assigns the less frequent allele as the minor allele that increases the risk when the odds ratio is greater than one.

We used 2x2xK (K = 11) Cochran-Mantel-Haenszel (CMH) statistics for the across-breed analysis. CMH is a standard test for a stratified case-control analysis. This was carried out in PLINK with the function --mh and with breed clusters defined with the --within function. Odds ratios and the respective 95% confidence intervals were automatically calculated by PLINK. The CMH test assumes homogeneity of the odds ratios between strata (breeds in our case), and violation of this assumption may lead to false positive associations [48]. Therefore, we used --homog function in PLINK to check if any of the SNPs demonstrating association in the CMH test, would violate the homogeneity assumption; all associated SNPs passed the homogeneity test. All permutation analyses (using 10,000 permutations) within and across breeds were executed with the max(T) permutation procedure in PLINK with the function -- mperm 10,000. We used a fixed seed (--seed 873,051,416) generated in Unix shell with “date +%N” to ensure reproducible results in all of the permutation analyses.

### STRING analysis

We used STRING (Search tool for retrieval for interacting genes/proteins) (V11.0) [15] to carry out a pathway analysis of the candidate gene sets, which we acquired from our association analyses. All genes from within 1 Mb from the variant that demonstrated significant association to CHD were listed and then used as an input for the STRING Multiple proteins search. The used candidate gene sets are listed in the Additional file 4. The STRING database was queried with 272 canine genes but seven genes (*TMEM244*, *STRA6L*, *FOXE1*, *RPS13*, *SERGEF*, *ESPNL*, and *FAB172B*) were not found. In addition, *U6* and *CNKSR3* were not recovered in the expected chromosome and were discarded. Thus, the STRING network for the positional candidate genes consisted 263 nodes (https://version-11-0.string-db.org/cgi/network.pl?networkId=5Sqk4IV9gi5b). We also performed an additional search with a combined set of neddylation pathway associated genes from Tables 3 and 14 genes highlighted in our previous studies (https://version-11-0b.string-db.org/cgi/network?networkId=bPwtieGk6WuV).

## Supplementary Information

**Additional file 1. **List of markers used in the validation study. 52 selected markers with their position in CanFam3.1 are listed. From the original publications the following details are presented: marker ID, associated phenotype, raw and corrected *P* value, and the cohort size and represented breeds. Regarding the current study, the breeds for which the QC failed are indicated as well as the ssID’s for the markers that passed the QC.

**Additional file 2.** Reference, alternative, major and minor alleles, and genotype frequencies for the 23 validated SNPs in the across-breed and within-breed studies.

**Additional file 3. **Quantile-Quantile plots of the within-breed association analyses. The image shows breed-wise comparisons of *P*-values (−log_10_) from a logistic regression model (logistic) or basic association analysis (X^2^ test; assoc).

**Additional file 4. **Candidate gene sets for the STRING (V11.0) pathway analysis. Two lists are provided: set of candidate genes from the across-breed association analysis (*N* = 41), and a set of candidate genes from the within-breed association analyses (*N* = 261). Total number of unique genes in the analyses is 272. Each of the listed genes are located maximum ±1 Mb from a SNP that demonstrated significant association to CHD in one of the analyses.

**Additional file 5. **Reactome pathway R-CFA-983169 on Class I MHC mediated antigen processing & presentation bridges the clusters of neddylation pathway associated genes (*ASB7, PSMC6, FBXO10, DCAF10, ASB4, ASB18, COPS8, UBE2F, ASB1, FBXW8, FBXO21, WSB2*) and the *NOX3/MMP2/MMP9/TRIO-*associated genes (*CYBA, MAPK14, MMP2, MMP9, NCF1, NCF2, NCF4, NOX3, NOXA1, NTN1, RAC1, TRIO, VCAM1*) from our previous studies on German Shepherds. Genes belonging to R-CFA-983169 are marked red. For a high resolution image and the original analysis, see: https://version-11-0b.string-db.org/cgi/network?networkId=bPwtieGk6WuV.

## Data Availability

The datasets generated and analysed during this study are available in the FIGSHARE repository: 10.6084/m9.figshare.11369511. The data was anonymised to protect the privacy of the dog owners.
