# Supplementary figures and images for: An across-breed validation study of 46 genetic markers in canine hip dysplasia
Source: BMC Genomics. 2021 Jan 21;22:68. doi: 10.1186/s12864-021-07375-x (PMC7818755; doi:10.1186/s12864-021-07375-x)

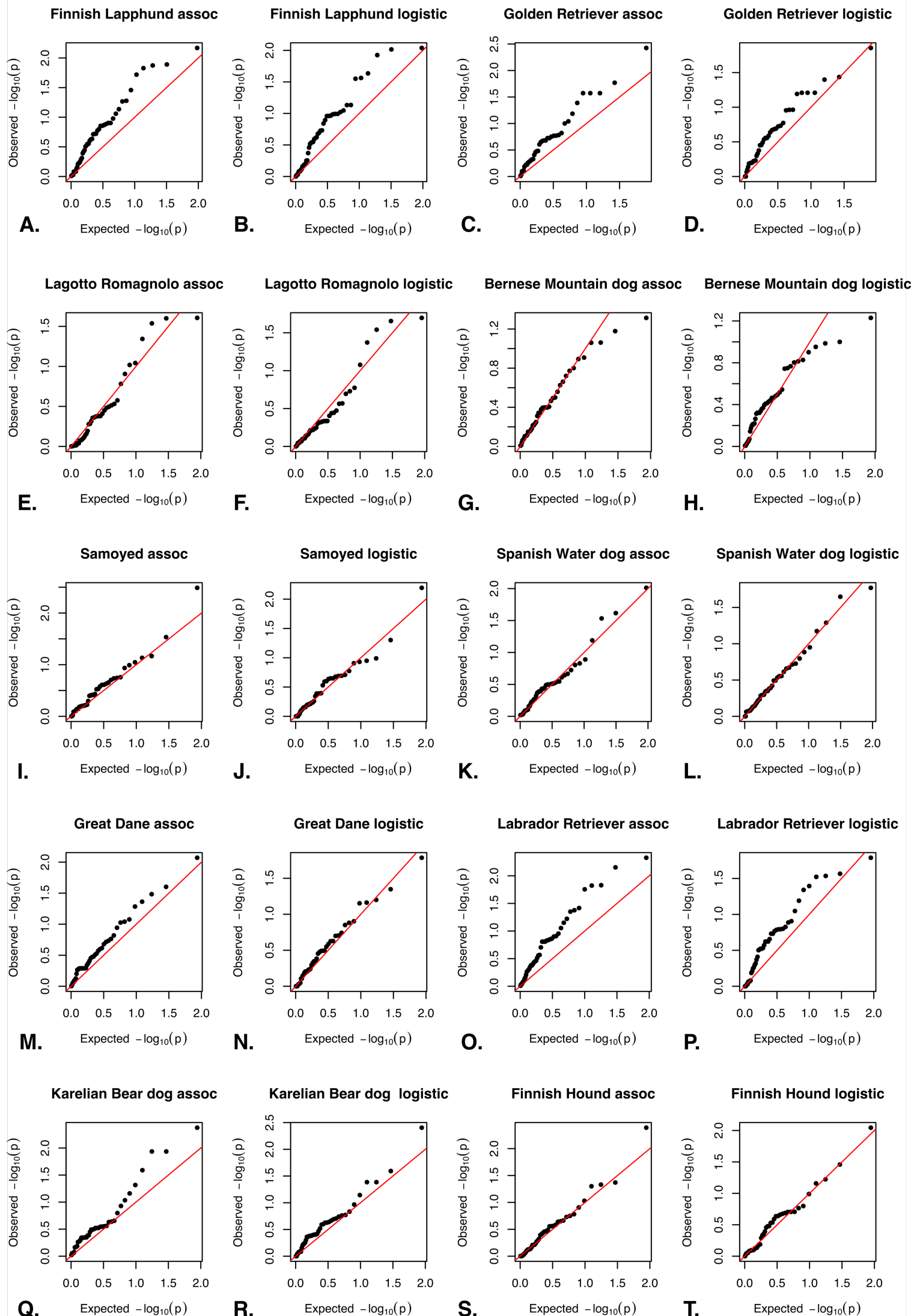

Supplement: Supplementary file 3 — Additional file 3. Quantile-Quantile plots of the within-breed association analyses. The image shows breed-wise comparisons of P-values (−log10) from a logistic regression model (logistic) or basic association analysis (X2 test; assoc). [file 12864_2021_7375_MOESM3_ESM.pdf]

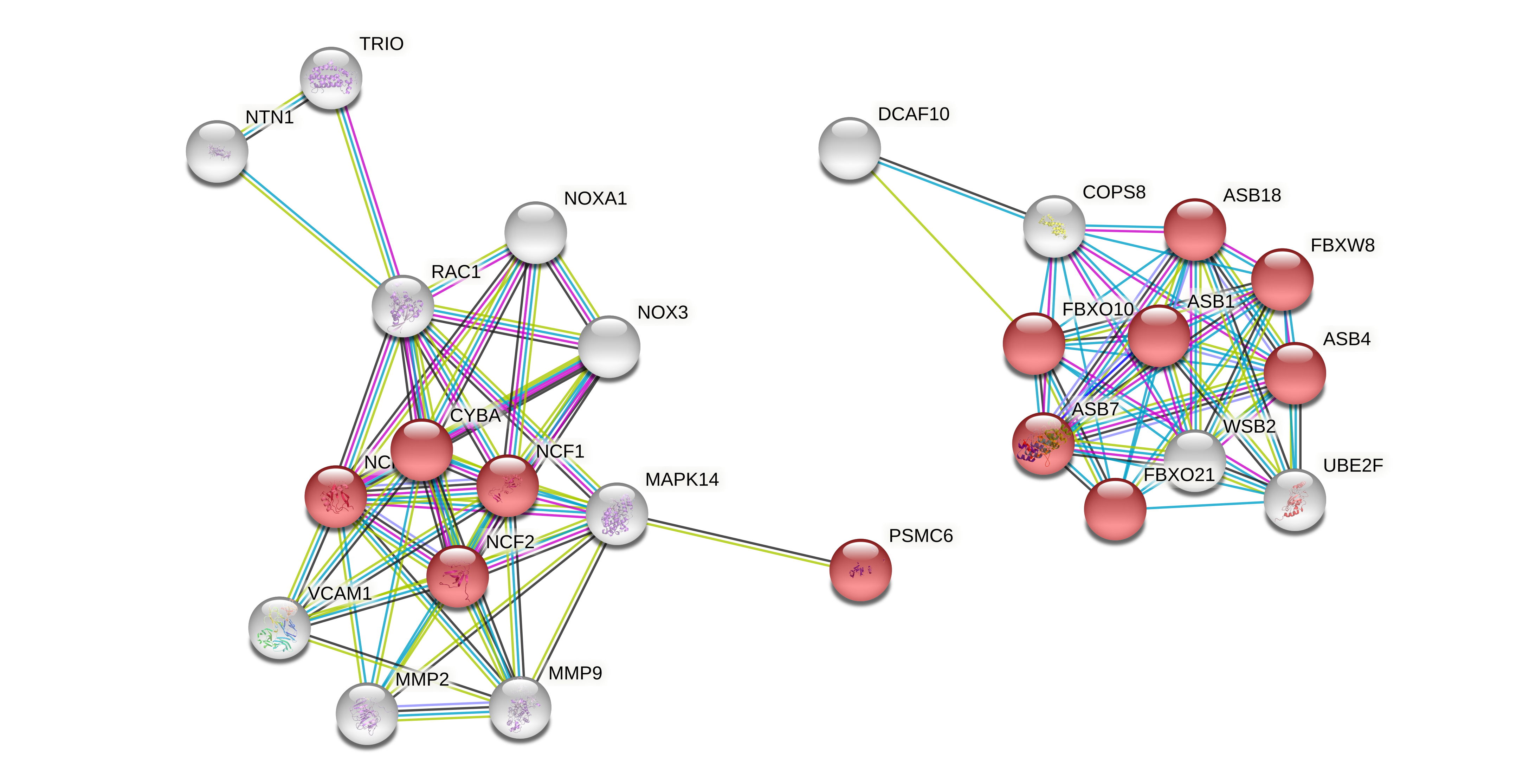

Supplement: Supplementary file 5 — Additional file 5. Reactome pathway R-CFA-983169 on Class I MHC mediated antigen processing & presentation bridges the clusters of neddylation pathway associated genes (ASB7, PSMC6, FBXO10, DCAF10, ASB4, ASB18, COPS8, UBE2F, ASB1, FBXW8, FBXO21, WSB2) and the NOX3/MMP2/MMP9/TRIO-associated genes (CYBA, MAPK14, MMP2, MMP9, NCF1, NCF2, NCF4, NOX3, NOXA1, NTN1, RAC1, TRIO, VCAM1) from our previous studies on German Shepherds. Genes belonging to R-CFA-983169 are marked red. For a high resolution image and the original analysis, see: https://version-11-0b.string-db.org/cgi/network?networkId=bPwtieGk6WuV. [file 12864_2021_7375_MOESM5_ESM.png]
